# Nanostructure and Corresponding Quenching Efficiency of Fluorescent DNA Probes

**DOI:** 10.3390/ma11020272

**Published:** 2018-02-09

**Authors:** Wenjuan Guo, Yanhong Wei, Zhao Dai, Guangping Chen, Yuanyuan Chu, Yifei Zhao

**Affiliations:** 1 State Key Laboratory of Separation Membranes and Membrane Processes, School of Environmental and Chemical Engineering, Tianjin Polytechnic University, Tianjin 300387, China; guowenjuan@tjpu.edu.cn (W.G.); weiyanhong1993@163.com (Y.W.); chuyuanyuan1982@163.com (Y.C.); zhaoyifeiaas@163.com (Y.Z.); 2 Department of Physiological Sciences, College of Veterinary Medicine, Oklahoma State University, Stillwater, OK 74078, USA; guangping.chen@okstate.edu

**Keywords:** fluorescent DNA probes, CdTe/SiO_2_ nanoparticles, Au nanoparticles, DNA hairpin template, nanostructure, quenching efficiency

## Abstract

Based on the fluorescence resonance energy transfer (FRET) mechanism, fluorescent DNA probes were prepared with a novel DNA hairpin template method, with SiO_2_ coated CdTe (CdTe/SiO_2_) core/shell nanoparticles used as the fluorescence energy donors and gold (Au) nanoparticles (AuNPs) as the energy acceptors. The nanostructure and energy donor/acceptor ratio in a probe were controlled with this method. The relationship between the nanostructure of the probes and FRET efficiency (quenching efficiency) were investigated. The results indicated that when the donor/acceptor ratios were 2:1, 1:1, and 1:2; the corresponding FRET efficiencies were about 33.6%, 57.5%, and 74.2%, respectively. The detection results indicated that the fluorescent recovery efficiency of the detecting system was linear when the concentration of the target DNA was about 0.0446–2.230 nmol/L. Moreover, the probes showed good sensitivity and stability in different buffer conditions with a low detection limit of about 0.106 nmol/L.

## 1. Introduction

Fluorescent DNA probes [1] are a kind of optical DNA biosensor [2] based on the fluorescence resonance energy transfer (FRET) mechanism [3]. FRET occurs when the electronic excitation energy of a donor chromophore is transferred to a nearby acceptor molecule via a through-space dipole–dipole interaction between the donor-acceptor pair within 1–10 nm distances. The energy donor molecule quickly jumps from the ground state to the electron excitation state. The field of fluorescent DNA probes improved considerably with the introduction of inorganic nanoparticles, such as quantum dots (QDs) [4,5] and gold (Au) nanoparticles (AuNPs) [6,7,8] as energy donors and acceptors [9], respectively. As a new kind of luminescent inorganic fluorophores, QDs are being widely used in chemical sensors [10], DNA detection [11], cell labeling [12], and imaging [13] because they have a broad and continuous excitation spectrum, a narrow size-tunable symmetric emission spectrum, and a high fluorescence quantum yield. AuNPs are widely used in gene delivery [14] and cell labeling [15] because they are bioinert, nontoxic, and readily synthesized and functionalized. In the field of fluorescent DNA biosensors, AuNPs are commonly used as energy acceptors because of their high extinction coefficient and wide absorption spectra.

The FRET-based DNA probe uses the hybridized DNA chains as scaffolds and detection union as a detection mechanism. The donor and acceptor are conjugated with complementary single-stranded DNA (ssDNA). After the hybridization between these ssDNA, the donor and acceptor must be within 1 and 10 nm of each other. According to the FRET mechanism, the fluorescence of the donor will be quenched, which results in the fluorescence sensor detecting low or no fluorescence background. The target ssDNA oligonucleotides, that have more complementary bases with one of the ssDNA of the probe, can open the double-stranded DNA (dsDNA) structure and hybridize with one of the conjugated ssDNA on the acceptor or donor surface. Therefore, the donor departs from the probe and the fluorescent intensity of the detection system recovers to some degree [16]. The QDs-DNA conjugates have been widely used for the detection of nucleic acids, including DNA, RNA, mRNA, miRNA, and other molecular ligands [17]. Zhang et al. developed a QDs-DNA-based single molecule FRET nanosensor that can detect a single point mutation in clinical samples [18]. Liang et al. demonstrated that mRNA QDs-DNA-based probes have a detection limit of less than 1 fmol [19]. Originally, the FRET-based probes were designed using the fluorescence “on/off” mechanism. In this way, the detection of the target DNA is measured by the changes in fluorescent intensities. However, the fluorescence background of QDs probes can interfere with the detection because the acceptors cannot totally quench the fluorescence of QDs donors. Therefore, some researchers designed QDs-DNA-based probes with a larger Stoke’s shift, to enable the multiplexed signal detection of the changes in fluorescent intensities, wavelength, and color [17]. Though this kind of FRET method avoids the fluorescent background of probes, a basic problem in this field is the lack of investigation. An inorganic nanoparticle can conjugate with more than one ssDNA on its surface [20,21]. Alivisatos et al. separated a DNA probe solution using gel electrophoresis, and reported that a QD could be conjugated with one to four AuNPs in one probe [21]. Therefore, the obtained probes were actually a mixture of complex nanostructures in terms of the ratio of donor/acceptor in one probe. However, few studies have focused on the relationship between the FRET efficiencies and nanostructures of inorganic nanoparticle-based probes.

The AuNPs that assemble using dsDNA scaffolds with different nanostructures have attracted much attention as building blocks [22,23] because thiol-terminated ssDNA (HS-DNA) easily self-assembles on the surface of AuNPs. Alivisatos et al. built pyramids with discrete and chiral nanostructures with dsDNA scaffolds and 5, 10, 15, and 20 nm AuNPs [21]. Dai et al. prepared a series of AuNPs assemblies with controllable, continuous, and discrete nanostructures with dimers, trimers, and tetramers when the bis(p-sulfonatophenyl)phenylphosphine (BSPP) acted as the ligand and the extended strands of ssDNA acted as the scaffolds [24]. However, few studies have used these to investigate the nanostructures of QDs-DNA-based probes. In previous work, a fluorescent DNA probe, with only one QD and one AuNP in the nanostructure, was prepared using an asymmetrical synthesis method with solid phase carriers, poly(*N*-isopropylacrylamide-*co*-divinylbenzene) microspheres, as the templates, and the corresponding quenching efficiency of probes was about 64.8% [16]. However, this method has more steps and the process, especially for the synthesis of carriers, is more complex. In addition, more probe nanostructures cannot be obtained with this method.

**Figure 1:**
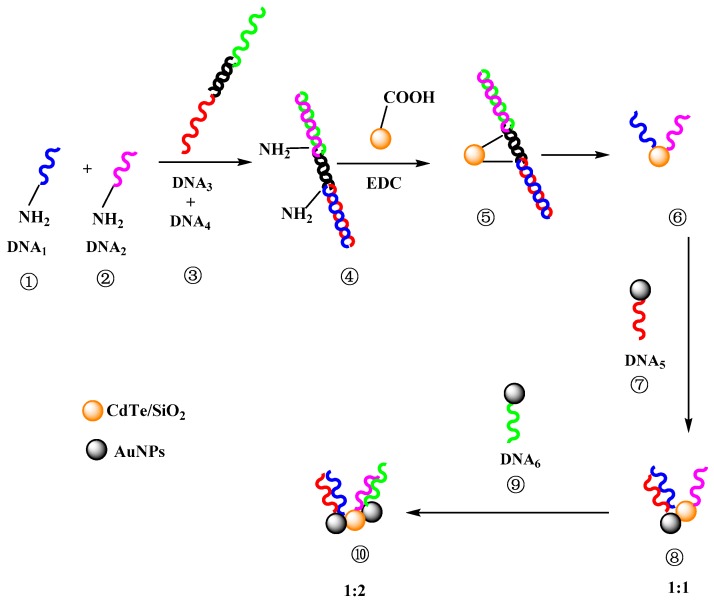
Controllable nanostructures of fluorescent DNA probes by DNA scaffolds (donor/acceptor ratio was 1:1 and 1:2, respectively).

In this paper, a simple method based on a dsDNA hairpin template for controlling the nanostructures of fluorescent DNA probes is presented. The amino-modified dsDNA hairpin template was hybridized with four ssDNA: ssDNA_1_, ssDNA_2_, ssDNA_3_, and ssDNA_4_. After the conjugation of the hairpin template and CdTe QDs, ssDNA_3_ and ssDNA_4_ were dehybridized and removed. Thus, the number and position of ssDNA on AuNPs or QDs could be controlled (Figure 1), and the relationship between the nanostructure and quenching efficiency was investigated.

## 2. Materials and Methods

### 2.1. Materials

Ethylenediaminetetraacetic acid, boric acid, chloroauric acid, sodium citrate, sodium chloride, hydrochloric acid, sodium borohydride, cyclohexanol, n-hexyl alcohol, aqueous ammonia, ethanol, acetone, isopropyl alcohol, succinic anhydride, *N*, *N*-dimethylformamide, and sodium hydroxide were analytical grade, purchased from Tianjin Kermel Chemical Reagent Co., Ltd. (Tianjin, China) Chloride cadmium and tellurium powder were analytical grade, purchased from Sinopharm Chemical Reagent Co., Ltd. (Shanghai, China) Mercaptopropionic acid (MPA), tetraethyl orthosilicate (TEOS), and 3-amino propyl diethoxymethyl silane (APTS) were analytical grade, while Tris (Tris, purity > 99.8%) was super grade, and all were purchased from Alfa Aesar Reagents Co., Ltd. (Shanghai, China) Dihydrate bis(p-sulfonyl phenyl) phenyl phosphine dipotassium (BSPP, purity > 97%) was purchased from J & K Scientific. (Beijing, China) Tetraethyl orthosilicate was analytical grade and was purchased from TCI Reagent Co. (Tokyo Japan) 1-ethyl-(3-dimethylaminopropyl) carbodiimide hydrochloride (EDC) was super grade, purchased from Aladdin Reagent Co., Ltd. (Shanghai, China) Single-stranded DNA (ssDNA) were purchased from Shanghai Invitrogen Biotechnique Co., Ltd. (Shanghai, China) and the base sequences were:

ssDNA_1_: 5′-NH_2_-TTT CTA TTC CTA CCA ATG TAG CGA CTA CCT CAG TTT TTT TTT TTT TTT TT-3′

ssDNA_2_: 5′-NH_2_-TTT CGA TCT AAT ACA GTT AGT TAG TAT ACG TGC TTT TTT TTT TTT TTT TT-3′

ssDNA_3_: 5′-CTG AGG TAG TCG CTA CAT TGG TAG GAA TAG GAT TGC ATG GGA TAC-3′

ssDNA_4_: 5′-GCA CGT ATA CTA ACT AAC TGT ATT AGA TCG GTA TCC CAT GCA ATC-3′

ssDNA_5_: 5′-TTT CTG AGG TAG TCG CTA CAT TGG TAG TTT TTT TTT-HS-3′

ssDNA_6_: 5′-TTT GCA CGT ATA CTA ACT AAC TGT ATT TTT TTT TTT-HS-3′

ssDNA_7_: 5′-HS-TTT CTA TTC CTA CCA ATG TAG CGA CTA CCT CAG TTT TTT TTT TTT TTT TT-3′)

ssDNA_8_: 5′-NH_2_-TTT CTA TTC CTA CCA ATG TAG CGA CTA CCT CAG TTT TTT TTT TTT TTT TT -3′

ssDNA_9_: 5′-TTT CTG AGG TAG TCG CTA CAT TGG TAG TTT TTT TTT-HS-3′

ssDNA_10_: 5′-TTT AAA AAA GCA CGT ATA CTA ACT AAC TGT ATT AGA TCG TTT-3′

ssDNA_11_: 5′-TTT TTT TTT ATG TAC GCG TCG GTC GGT CAC GCC GAG CTA TTT-3′

ssDNA_12_: 5′-TTT AAA AAA GCA CGT ATA ATA ACT AAC TGT ATT AGA TCG TTT-3′

### 2.2. Preparation of Carboxyl-Modified CdTe/SiO_2_ (CdTe/SiO_2_-COOH)

CdTe QDs as energy donors were prepared in an aqueous solution according to the reference work with 3-mercaptopropionic acid (MPA) used as the stabilizer [25]. A total of 57.0 mg of NaBH_4_, 23.9 mg of Te powder, and 4 mL of double-distilled water were added into a 5-mL injector and stored at 4 °C for about 12 h to obtain NaHTe. Then, 85.2 mg of CdCl_2_, 78 μL of MPA, and 100 mL of double-distilled water were introduced into a 100-mL round-bottom flask with N_2_ protection. The pH of the mixture was adjusted to 9.1 with 2 M of NaOH solution under stirring. The freshly prepared NaHTe solution was rapidly injected into the reaction mixture with N_2_ protection under constant stirring for about 30 min. The reaction mixture was heated from ambient temperature until it boiled at 100 °C. The reflux time for obtaining the CdTe QDs was 3 h.

CdTe/SiO_2_ fluorescent nanoparticles were synthesized according to the reverse microemulsion method [26]. A total of 2.5 mL of CdTe QDs μL aqueous solution and 1.25 mL of aqueous ammonia (25 wt %) were introduced into a liquid system containing 37.5 mL of cyclohexane, 9 mL of n-hexanol, and 8.85 mL of Triton X-100 under stirring for 30 min. Then, 100 μL of tetraethyl orthosilicate (TEOS) was added into the reaction system with vigorous stirring. The silica growth was completed in the dark at room temperature for 24 h. The resultant nanoparticles were isolated from the microemulsion using acetone and an ultracentrifuge, and the precipitate of the CdTe/ SiO_2_ composite particles was sequentially washed with 1-butanol, iso-propanol, ethanol, and water to remove the Triton X-100 and unreacted molecules.

The CdTe/SiO_2_ nanoparticles were modified by using a silane coupling agent 3-aminopropyl triethylsilane (APTES), and succinic anhydride to create carboxyl groups on the surface (CdTe/SiO_2_-COOH nanoparticles). A total of 0.2 g of CdTe/SiO_2_ and 19 mL of ethanol were added into two 50-mL round bottom flasks under ultrasound for 1 h. Then, a certain amount of APTES was added into the reaction system with stirring at 25 °C for 5 h. Then, the purified nanoparticles, 30 mL of *N*, *N*-dimethylformamide (DMF), and 0.5 g of succinic anhydride were mixed under ultrasound for 1 h and then stirred at ambient temperature for 12 h. The CdTe/SiO_2_-COOH nanoparticles were washed three times with ethanol by centrifugation and dried under a vacuum.

### 2.3. Preparation of DNA Hairpin Templates

NH_2_-dsDNA hairpin templates were hybridized from ssDNA_1_, ssDNA_2_, ssDNA_3_, and ssDNA_4_. A total of 0.1 μmol of the above ssDNA were mixed with 1 mL of Tris-HCl buffer solution (0.05 M Tris-HCl containing 50 μL of BSPP (10 mg/mL) and 2 M of NaCl) when the molar ratio of DNA_1_:DNA_2_:DNA_3_:DNA_4_ was 1:1:1:1 in a 90 °C water bath for 10 min, and then cooled down to 30 °C at a rate of −3 °C/3 min. The DNA hairpin template solution was added to 0.5 mL of 0.5× TBE buffer (45 mM Tris-HCl, 45 mM boric acid, 1 mM EDTA) containing 10 mg/mL BSPP and 50 mM NaCl, then stored and at 4 °C.

The HS-dsDNA hairpin templates were prepared with ssDNA_3_, ssDNA_4_, ssDNA_5_, and ssDNA_6_ using the same method.

### 2.4. Conjugation of DNA Template and CdTe/SiO_2_ (CdTe/SiO_2_-dsDNA Hairpin) and Dehybridization (CdTe/SiO_2_-ssDNA)

CdTe/SiO_2_-COOH fluorescent nanoparticles (5 mg) were dispersed in 1 mL Tris-HCl buffer (0.01 M Tris-HCl containing 20 mM NaCl, pH = 7.2) under stirring. Then, DNA hairpin template (1 mL, 100 μmol/L) of Tris-HCl buffer solution (0.05 M, pH = 8.0) and 1-ethyl-3-(3-dimethylaminopropyl propyl) carbodiimide hydrochloride (EDC, 22 μL, 0.01 M) were added into the nanoparticle dispersion. The mixture was incubated at 25 °C for 40 h under shaking. The CdTe/SiO_2_-DNA hairpin complexes were separated by ultracentrifugation, and washed with Tris-HCl buffer solution three times. The purified complexes were resuspended in 2 mL Tris-HCl buffer and stored at 4 °C for 24 h.

For the dehybridization of the template on the CdTe/SiO_2_, the sediment was incubated at 40 °C for 15 min. The ssDNA_3_ and ssDNA_4_ in the dsDNA template were released from the surface of the CdTe/SiO_2_ nanoparticles and removed from the suspension together with the supernatant after centrifugation under the same conditions described above. The dehybridization process was repeated three times. The resulting sediment, after the repetition of the dehybridization, was resuspended in a hybridization buffer (0.5 mL of 0.5× TBE buffer containing 0.25 mg/mL BSPP and 50 mM NaCl).

### 2.5. Conjugation of 40-nm AuNPs with ssDNA (AuNPs-ssDNA)

Fren’s method was used to obtain AuNPs when the citrate was used as the reduction [24]. HAuCl_4_ (1 mL, 1%) was added into 100 mL of double-deionized water and fluxed until boiling. Then, 1% trisodium citrate solution was quickly added into this mixture with strong stirring for 15 min. After the color of reaction system turned wine-red, the reaction was stopped and cooled to room temperature. AuNPs coated with BSPP had a high stability in buffer solutions [24]. The AuNP (500 μL, 16 or 40 nm) suspension was mixed with 100 μL of BSPP solution (50 mg/mL in water) in a microtube and incubated at 50 °C for 1 h. The suspension was then centrifuged, and washed with 0.5× TBE including BSPP (1 mg/mL) buffer solution for three times. The resulting BSPP stabilized AuNPs were resuspended in 1 mL of 0.5× TBE including BSPP (1 mg/mL) buffer solution and stored at 4 °C. The AuNP concentration in the suspensions was estimated using the ultraviolet (UV)-vis spectra of the AuNPs.

AuNPs-ssDNA conjugations were prepared using the traditional method. The AuNPs were conjugated with thiolated ssDNA (ssDNA_5_ or ssDNA_6_), by mixing 200 μL of BSPP-coated AuNPs, 8 μL of 0.5× TBE containing 1.4 M NaCl, 0.1 μmol of ssDNA in 0.5× TBE containing 1 mg/mL BSPP, and 10 mM NaCl. The mixture was incubated under shaking at 25 °C for 24 h. The ssDNA-conjugated AuNPs were separated using ultracentrifugation, and washed with the diluted BSPP solution three times. Finally, the sediment was resuspended in a 0.2 mg/mL BSPP solution.

The synthesis of the 40-nm AuNPs-ssDNA (Au40-ssDNA) conjugation using the mercapto-dsDNA hairpin template was the same as that described in Section 2.4.

### 2.6. Preparation and Detection of Fluorescent DNA Probes

The resultant suspensions prepared in Section 2.4 and Section 2.5 were mixed at different ratios. CdTe/SiO_2_-ssDNA and AuNPs-ssDNA were hybridized in a buffer solution of 20 mM Tris-HCl, 50 mM NaCl, and 5 mM MgCl_2_ (pH = 8.0) at 37 °C, for 12 h under shaking. For detection, an amount of target ssDNA was introduced into the detection solution under shaking for 12 h. The fluorescence spectra of the probe system were measured with an F-7000 Luminescence Spectrometer (Hitachi, Japan). According to Foster’s theory, the FRET efficiency (quenching efficiency, *Q_e_*) can be measured experimentally and is commonly defined as [16]:

(1) $$Q_{e}=(1-\frac{F_{DA}}{F_{D}})\times 100\%$$

where *F_DA_* is the integrated fluorescence intensity of the donor in the presence of the acceptor(s) and *F_D_* is the integrated fluorescence intensity of the donor alone (no acceptors present).

When the complementary DNA was present, the hybridized structure of probes opens and the fluorescent intensity of detection system recovers. The fluorescent recovery efficiency (*F_r_*) is given as:

(2) $$F_{r}=(\frac{F-F_{0}}{F_{0}})\times 100\%$$

where *F*_0_ is the fluorescent intensity of the DNA probe, and *F* is the fluorescent intensity of the DNA probe with target ssDNA.

## 3. Results and Discussion

### 3.1. Synthesis and Modification of CdTe/SiO_2_ Composite Nanoparticles

**Figure 2:**
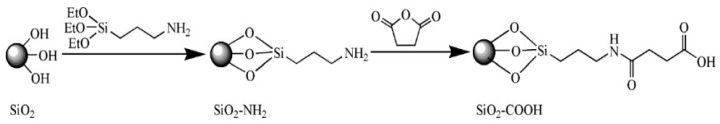
Surface modification mechanism of CdTe/SiO_2_ fluorescent nanoparticles.

The aqueous synthesis of CdTe QDs resulted in a small particle size of about 2–3 nm [25]. The coat of SiO_2_ increases the particle size for coverage by the DNA hairpin template. The SiO_2_-coated CdTe (CdTe/SiO_2_) was prepared by the hydrolysis of tetraethyl orthosilicate (TEOS) in a reverse micro-emulsion, with ammonia and CdTe QDs in the aqueous phase, cyclohexane and n-hexanol in the oil phase, and the surfactant TritonX-100 as the emulsifier. For the reaction with the amino groups of the DNA template, the CdTe/SiO_2_ nanoparticles were modified with carboxyl surface groups using APTES and succinic anhydride (Figure 2).

**Figure 3:**
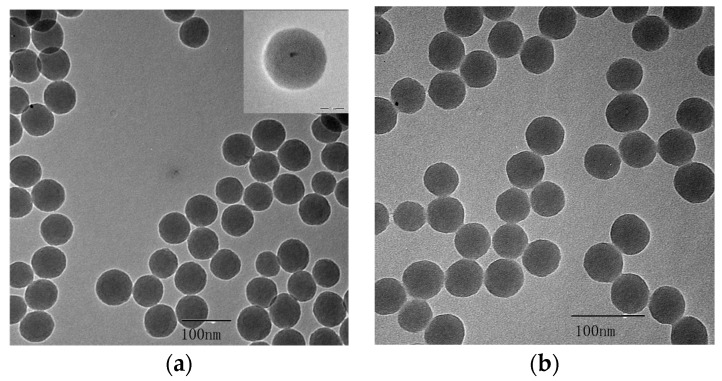
TEM of (**a**) CdTe/SiO_2_ and (**b**) carboxyl-modified CdTe/SiO_2_ particles.

The transmission electron microscopy (TEM) results for CdTe/SiO_2_ indicated that the CdTe/SiO_2_ fluorescent nanoparticles were about 50 nm in diameter with a round and smooth surface, and the particle size had no obvious changes after modification (Figure 3).

**Figure 4:**
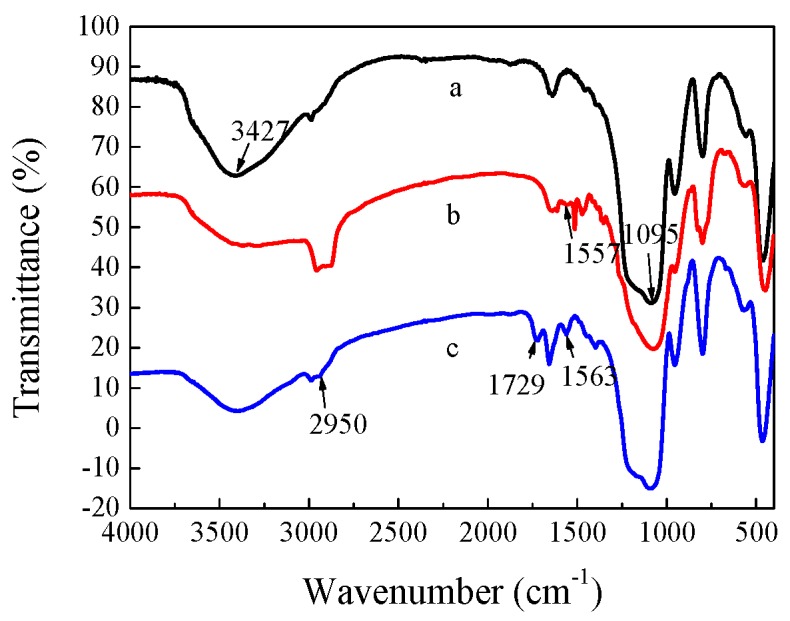
FT-IR of (**a**) CdTe/SiO_2_; (**b**) CdTe/SiO_2_-NH_2_; (**c**) CdTe/SiO_2_-COOH particles.

The Fourier transform infrared (FT-IR) spectra of CdTe/SiO_2_ particles before and after modification are shown in Figure 4. The original CdTe/SiO_2_ fluorescent nanoparticles had strong absorption peaks of Si-OH at 3427 and 945 cm^−1^, as well as the Si-O-Si absorption peak at 1095 cm^−1^ (Figure 4a). The absorption peak at 1563 cm^−1^ indicated that the amino groups were introduced to the surface of the CdTe/SiO_2_ particle (Figure 4b), and the peak at 1729 cm^−1^ was the carboxyl groups of CdTe/SiO_2_-COOH particles (Figure 4c), indicating that the modification was successful.

### 3.2. Carriable ssDNA Number on AuNPs and CdTe/SiO_2_ Particles

**Figure 5:**
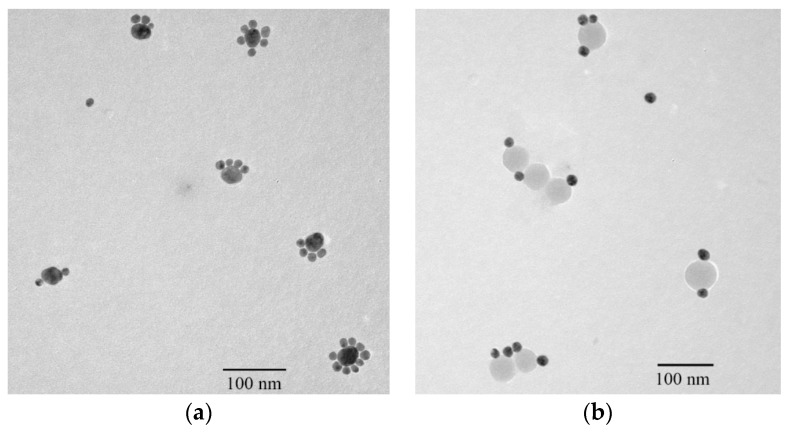
Nanostructures of (**a**) Au nanoparticles (AuNPs) self-assemblies and (**b**) fluorescent DNA probes by the traditional method.

If ssDNA is freely conjugated into inorganic particles in an aqueous solution using the traditional method, the number of ssDNA on a particle cannot be controlled [20,21]. Keating et al. reported that the curvature of a AuNP considerably influenced the possible number of ssDNA on its surface. Small diameter AuNPs carry a lower number of thiolated single-stranded ssDNA strands [27]. In this work, 16 and 40-nm AuNPs (Au_16_ and Au_40_) were used to investigate the possible ssDNA number on AuNPs when they were conjugated with two complementary ssDNA: ssDNA_7_ and ssDNA_9_. The possible ssDNA numbers on AuNPs results are shown in Figure 5, indicating that thiol-terminated ssDNA (HS-DNA) could be easily self-assembled on the surface of Au_40_ with a Au-S bond. Therefore, one Au_40_ could carry about two to nine ssDNA_7_, while one Au_16_ could carry only one ssDNA_9_ (Figure 5a), which confirmed Keating’s curvature theory of AuNPs [27]. Moreover, when the CdTe/SiO_2_ nanoparticles (with ssDNA_8_) and Au_16_ (with ssDNA_9_) were used to prepare fluorescent DNA probes using the same method, the results showed that the carriable ssDNA number was also one to three (Figure 5b), which indicated that the conjugation of NH_2_-ssDNA with CdTe/SiO_2_ was more difficult than that of HS-ssDNA with AuNPs.

### 3.3. Controllable Nanostructures of DNA Probes by DNA Hairpin Template

**Figure 6:**
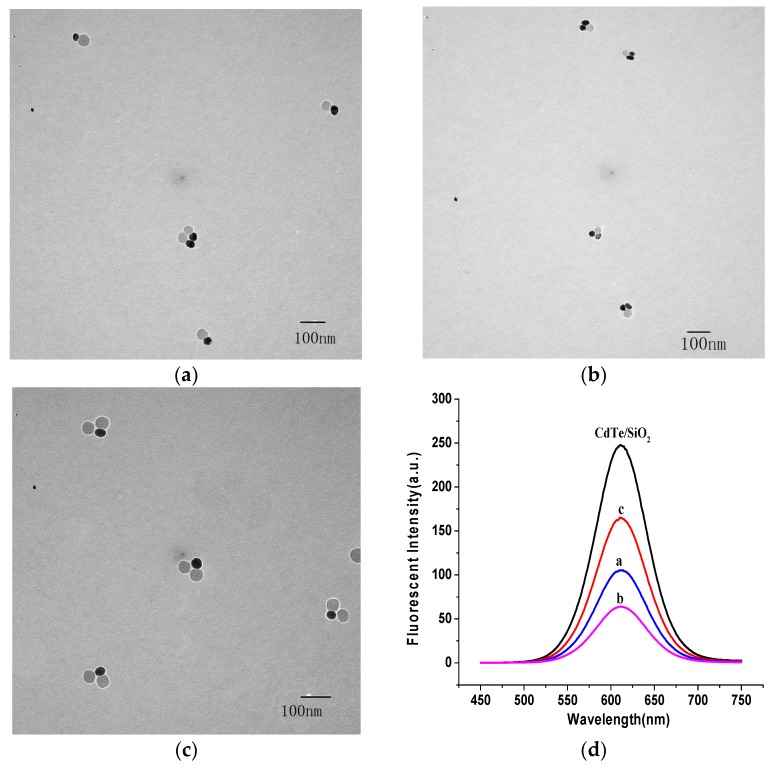
Different nanostructure of fluorescent DNA probes by DNA hairpin template. (**a**) 1:1; (**b**) 1:2; (**c**) 2:1; and (**d**) corresponding fluorescent emission spectra.

For the investigation and control of the nanostructures of a DNA probe, CdTe/SiO_2_ nanoparticles, as energy donors, were conjugated with a NH_2_-dsDNA hairpin template (hybridization by ssDNA_1_, ssDNA_2_, ssDNA_3_, and ssDNA_4_). After the release of ssDNA_3_ and ssDNA_4_ from the conjugation, only one ssDNA_1_ and ssDNA_2_ remained on the particle surface (Figure 1) because the maximum ssDNA number on CdTe/SiO_2_ nanoparticles is three. Therefore, because ssDNA_5_ cannot hybridize with ssDNA_2_, the CdTe/SiO_2_-ssDNA_1_-ssDNA_2_ conjugations could only hybridize with one ssDNA_5_ on Au_40_ and form the conjugations of CdTe/SiO_2_-dsDNA-Au_40_, the fluorescent DNA probes, into a 1:1 donor/acceptor (CdTe/SiO_2_:Au_40_) structure (Figure 6a). If the Au_40_-ssDNA_6_ conjugations were added into this preparation system, the fluorescent DNA probes could have a 1:2 donor/acceptor ratio in the nanostructure (Figure 6b) because ssDNA_6_ was only complementary to ssDNA_2_. Through the same process, when the HS-DNA hairpin template (hybridization by ssDNA_3_, ssDNA_4_, ssDNA_5_, and ssDNA_6_) was used, the fluorescent DNA probes in a 2:1 donor/acceptor (CdTe/SiO_2_:Au_40_) radio could be prepared (Figure 6c).

Due to the dsDNA hybridization of probes, the distance between the energy donor, CdTe/SiO_2_, and energy acceptor, AuNPs, is close enough to result in the FRET effect (quenching effect) and a decrease in the fluorescence intensity of the DNA probes. Though some investigations about nanostructures of DNA probes based on inorganic nanoparticles have been completed [21], research into the relationship between nanostructures and the corresponding quenching effect is limited. In this study, the nanostructures of CdTe/SiO_2_-dsDNA-Au_40_ probes were controlled with hairpin scaffolds and the corresponding quenching efficiency (Q_e_) was 33.6%, 57.5%, and 74.2% when the donor/acceptor ratios of the probes were 2:1, 1:1, and 1:2, respectively (Figure 6d).

### 3.4. Detection for Target DNA

**Figure 7:**
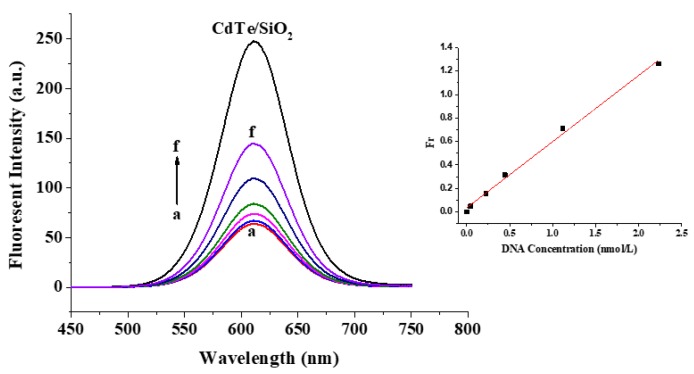
Detection of target DNA with different concentrations and the corresponding working curve: (**a**) 0 nmol/L; (**b**) 0.0446 nmol/L; (**c**) 0.223 nmol/L; (**d**) 0.446 nmol/L; (**e**) 1.115 nmol/L; and (**f**) 2.230 nmol/L.

The detection of the DNA probe as a biosensor for completely complementary DNA (ssDNA_10_) as the target was investigated when the DNA probe had a 1:2 donor/acceptor ratio in the nanostructure (Figure 7). The fluorescent intensities of the detection system gradually increased with the increase in ssDNA_10_ concentration. When the ssDNA_10_ concentration range was 0.0446 to 2.230 nmol/L, the corresponding linear regression equation was *F_r_* = 0.034 + 0.565c with an R^2^ of 0.995, where *F_r_* and *c* are the fluorescent increase efficiency and ssDNA_10_ concentration, respectively. The detection limit for completely complementary DNA was about 0.106 nmol/L.

**Figure 8:**
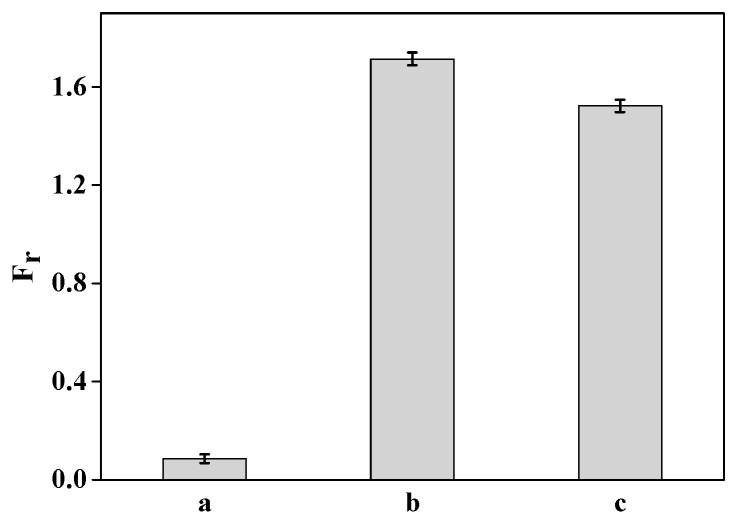
Selectivity of DNA probe when the target DNA concentration was 1.115 nmol/L. (**a**) Completely non-complementary DNA; (**b**) completely complementary DNA; and (**c**) a mismatched base-pair DNA.

The specificity of the DNA probe was investigated when the completely non-complementary DNA (ssDNA_11_), completely complementary DNA (ssDNA_10_), and a mismatched base-pair DNA (ssDNA_12_) were the target DNA (Figure 8). The results indicated that when the completely non-complementary ssDNA_11_ was the target, the fluorescent intensity slightly increased by about 8.6% (Figure 8a); whereas when the completely complementary ssDNA_10_ and the mismatched base-pair ssDNA_12_ were the targets, the increase in fluorescent intensities were 171.4% and 152.3% (Figure 8b,c), respectively, when the DNA concentration was 1.115 nmol/L. Therefore, this DNA probe can be used to detect whether a single point mutation has occurred on the target DNA.

**Figure 9:**
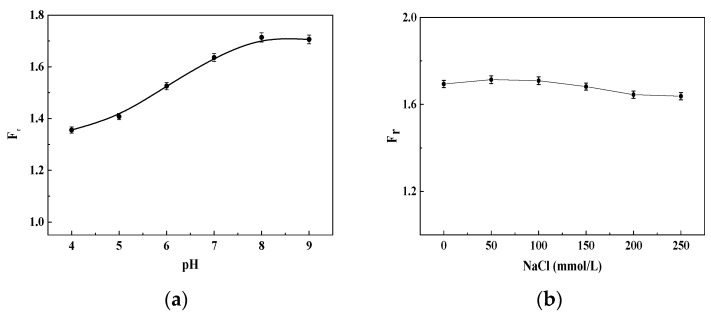
The fluorescence responses of the DNA detection system in different buffer conditions with 20 mmol/L Tris-HCl and 1.115 nmol/L ssDNA_10_. (**a**) pH; (**b**) NaCl concentration.

Both the fluorescence of the donors and the hybridized structure of dsDNA in probes could change under different detection conditions [24,28]. Therefore, the stability of this fluorescent DNA probe system was investigated for different buffer conditions and pH values (Figure 9). The results indicated that the FRET recovery efficiency (*F_r_*) of the detection system reached a maximum when the pH value was 8 and the ssDNA_10_ concentration was 1.115 nmol/L (Figure 9a). Therefore, the optimal pH for detection was about 8.0. The effect of NaCl on *F_r_* in the binding buffer solution indicated that the *F_r_* did not obviously change with different NaCl concentrations (Figure 9b) because the BSPP could improve the stability of inorganic nanoparticle-based DNA probes [24].

## 4. Conclusions

A DNA hairpin template method was presented in this paper for the preparation of fluorescent DNA probes and to control the nanostructure (energy donor/acceptor ratio) of probes, when CdTe/SiO_2_ fluorescent nanoparticles were the energy donors and AuNPs were the energy acceptors. A series of DNA probes with different donor/acceptor ratios, 2:1, 1:1, and 1:2, were obtained and the corresponding FRET efficiency of the probes were 33.6%, 57.5%, and 74.2%, respectively. Moreover, this probe showed good sensitivity and specificity under different buffer conditions.
